# The effects of metformin in the treatment of osteoarthritis: Current perspectives

**DOI:** 10.3389/fphar.2022.952560

**Published:** 2022-08-23

**Authors:** Yanhong Song, Ziyi Wu, Ping Zhao

**Affiliations:** Department of Anesthesiology, Shengjing Hospital of China Medical University, Shenyang, China

**Keywords:** metformin, osteoarthritis, articular cartilage degeneration, AMPK, chondrocyte

## Abstract

Osteoarthritis is a chronic and irreversible disease of the locomotor system which is closely associated with advancing age. Pain and limited mobility frequently affect the quality of life in middle-aged and older adults. With a global population of more than 350 million, osteoarthritis is becoming a health threat alongside cancer and cardiovascular disease. It is challenging to find effective treatments to promote cartilage repair and slow down disease progression. Metformin is the first-line drug for patients with type 2 diabetes, and current perspectives suggest that it cannot only lower glucose but also has anti-inflammatory and anti-aging properties. Experimental studies applying metformin for the treatment of osteoarthritis have received much attention in recent years. In our review, we first presented the history of metformin and the current status of osteoarthritis, followed by a brief review of the mechanism that metformin acts, involving AMPK-dependent and non-dependent pathways. Moreover, we concluded that metformin may be beneficial in the treatment of osteoarthritis by inhibiting inflammation, modulating autophagy, antagonizing oxidative stress, and reducing pain levels. Finally, we analyzed the relevant evidence from animal and human studies. The potential of metformin for the treatment of osteoarthritis deserves to be further explored.

## Introduction

Metformin (C4H11N5) is a widely known oral hypoglycemic agent in the biguanide category. It is derived from galegine, a natural product from the plant Galega officinalis (Thomas and Gregg, 2017). Metformin has been in clinical use for more than 60 years since it was first introduced in 1957. In the 1960s, studies found that phenformin, one of the biguanide drugs, increased the risk of cardiovascular death and lactic acidosis, which led to questions about the clinical application of metformin (Keller and Berger, 1983). However, long-term findings confirmed the glucose-lowering effects and cardiovascular benefits of metformin ((UKPDS) Group, 1998). In 1994, the FDA approved metformin for the treatment of patients with type 2 diabetes (Alušík and Paluch, 2015). Nowadays, it has been widely used in clinical practice, and its glucose-lowering mechanism involves inhibition of hepatic gluconeogenesis, promotion of glucose uptake and utilization by peripheral tissues (muscle and fat), inhibition of glucose uptake by intestinal wall cells, improvement of insulin sensitivity, and increase of glucagon-like peptide-1 (GLP-1) level, etc. (Rena et al., 2017; Foretz et al., 2019; He, 2020). New insights suggest that metformin is not only hypoglycemic in the endocrine system, but also protective in cardiovascular diseases, skeletal muscular diseases, reproductive diseases, cancer, and aging (Romero et al., 2017; Lv and Guo, 2020).

As the global population ages in the 21st century, osteoarthritis (OA) is becoming a major threat to human health. Epidemiological research has shown that the global population of people with osteoarthritis has exceeded 350 million (Safiri et al., 2020), and it is expected that the prevalence of osteoarthritis in people over 45 years of age may increase to 29.5% by 2032 (Hunter and Bierma-Zeinstra, 2019). Osteoarthritis is a degenerative joint disease characterized by pain, joint stiffness, and joint dysfunction. The pathology commonly involves degeneration and loss of articular cartilage, formation of bony redundancies at the joint edges, and reactive subchondral bone proliferation. The incidence and severity of the disease are often closely related to aging (Loeser et al., 2016). In addition, increased joint loading related to obesity, joint wear due to uncoordinated exercise, lack of muscle support caused by lack of exercise, joint damage linked to infection and inflammation, age-related loss of bone density, and changes in sex hormone levels may all be closely associated with the development of the disease (Palazzo et al., 2016). In the early stages of the disease, patients may be asymptomatic or may only show pain and minor functional impairment, but as the disease progresses, disability and other negative consequences may occur, which will seriously affect the quality of life of patients. Bone and joint diseases are becoming a health threat alongside cardiovascular diseases and cancer. However, despite many efforts, there is still a lack of effective treatment for osteoarthritis, and the main objective of treatment is to reduce pain and slow down disease progression (Taruc-Uy and Lynch, 2013). AMPK, an AMP-activated protein kinase, is an evolutionarily highly conserved cellular energy regulator that consists of a heterotrimeric structure of catalytic subunit *α* and regulatory subunits *β* and *γ*. Research evidence suggests that AMPK activity in chondrocytes is critical for maintaining joint homeostasis and that downregulation of AMPK activity is associated with the progression of osteoarthritis (Petursson et al., 2013; Zhao et al., 2014; Zhou et al., 2017). Moreover, AMPK may be a new therapeutic target for pain, and its regulation may be effective in treating chronic pain-related diseases (Asiedu et al., 2016). The activation of AMPK by metformin does not result from direct action, but rather through inhibition of complex I of the mitochondrial respiratory chain, which leads to an increase in the AMP levels. The new findings demonstrate that metformin can also activate AMPK independently via the lysosomal pathway (Agius et al., 2020). Given the role of AMPK in pain signaling, chondrocyte metabolism, and the fact that metformin is a potent AMPK activator, metformin may be a promising drug for preventing and slowing the progression of osteoarthritis.

## Molecular mechanisms of metformin action

Metformin can act through AMPK-dependent and AMPK-independent pathways, mainly involving inhibition of mitochondrial respiration, lysosomal-related pathways, and inhibition of expression of gluconeogenesis-related enzymes (Rena et al., 2017; Agius et al., 2020). Metformin specifically inhibits complex I of the mitochondrial respiratory chain, leading to an increase in the AMP: ATP/ADP: ATP ratio in cells. Changes in the NAD^+^: NADH ratio may also result from the inhibition of the mitochondrial respiratory chain and contribute to the effects of metformin (Alshawi and Agius, 2019). AMPK is a heterotrimer composed of three subunits, *α*, *β*, and *γ*, and each subunit is divided into multiple domains. Activation of AMPK by AMP is caused by a complementary mechanism (antagonized by ATP). AMP can directly bind to AMPK and exert an effect in the following ways: 1) increasing the rate at which LKB1 phosphorylates Thr172; 2) decreasing the rate at which protein phosphatases dephosphorylate Thr172 and 3) binding to theγ subunit of AMPK (allosterically activated) (Fyffe et al., 2018). Hawley et al. found that metformin did not activate AMPK in mutant cells that were insensitive to AMP changes but capable of expressing AMPK, which supported the idea that metformin could work through increasing cellular AMP (Hawley et al., 2010). However, subsequent studies have added that metformin can activate AMPK through the lysosomal pathway and not just by inhibiting the mitochondrial respiratory chain. LKB1 in hepatocytes can be co-transported to the lysosomal surface by the scaffolding protein Axin and thus play a role in AMPK activation (Agius et al., 2020). In a recent study in mammalian liver primary cells, AMPK was activated after drug administration without significant changes in the ATP, AMP, and ADP ratios. This suggests that low doses of metformin (5 μM) can activate AMPK independent of AMP. This study proposes that metformin inhibits the ATP6AP1 protein of the lysosomal V-ATPase complex by binding to progerin-enhancing factor-2 (PEN2), which in turn mediates lysosomal activation of AMPK, inhibition of mTOR (Ma et al., 2022). Furthermore, metformin may act through an AMPK-independent pathway. It has been proposed that metformin can inhibit hepatic gluconeogenesis in a manner independent of LKB1 and AMPK, and the same inhibition of glucose production by metformin was observed in mouse hepatocytes lacking the catalytic subunit of AMPK (Foretz et al., 2010). AMP might have an additional AMPK-independent effect and a possible mechanism to explain the above phenomenon is that elevated AMP reduces the production of c-AMP and inhibits the expression of gluconeogenesis-related enzymes such as fructose-1,6-bisphosphatase (Miller et al., 2013; Rena et al., 2017). Additionally, the mechanism of action of metformin is related to the dosage (He and Wondisford, 2015). High concentrations of metformin (∼5 mM) inhibited respiratory chain complex 1 and subsequently led to an increase in the AMP/ATP ratio. However, low concentrations may not be sufficient to increase the AMP/ATP and ADP/ATP ratios but can suppress dibutyryl-cAMP-stimulated gluconeogenic gene expression (Maiuri et al., 2007). Overall, drug dose and cell type may lead to metformin acting through different mechanisms. Whether other pathways might mediate the action of metformin remains to be further investigated in the future.

## Mechanism of metformin action in osteoarthritis

**FIGURE 1:**
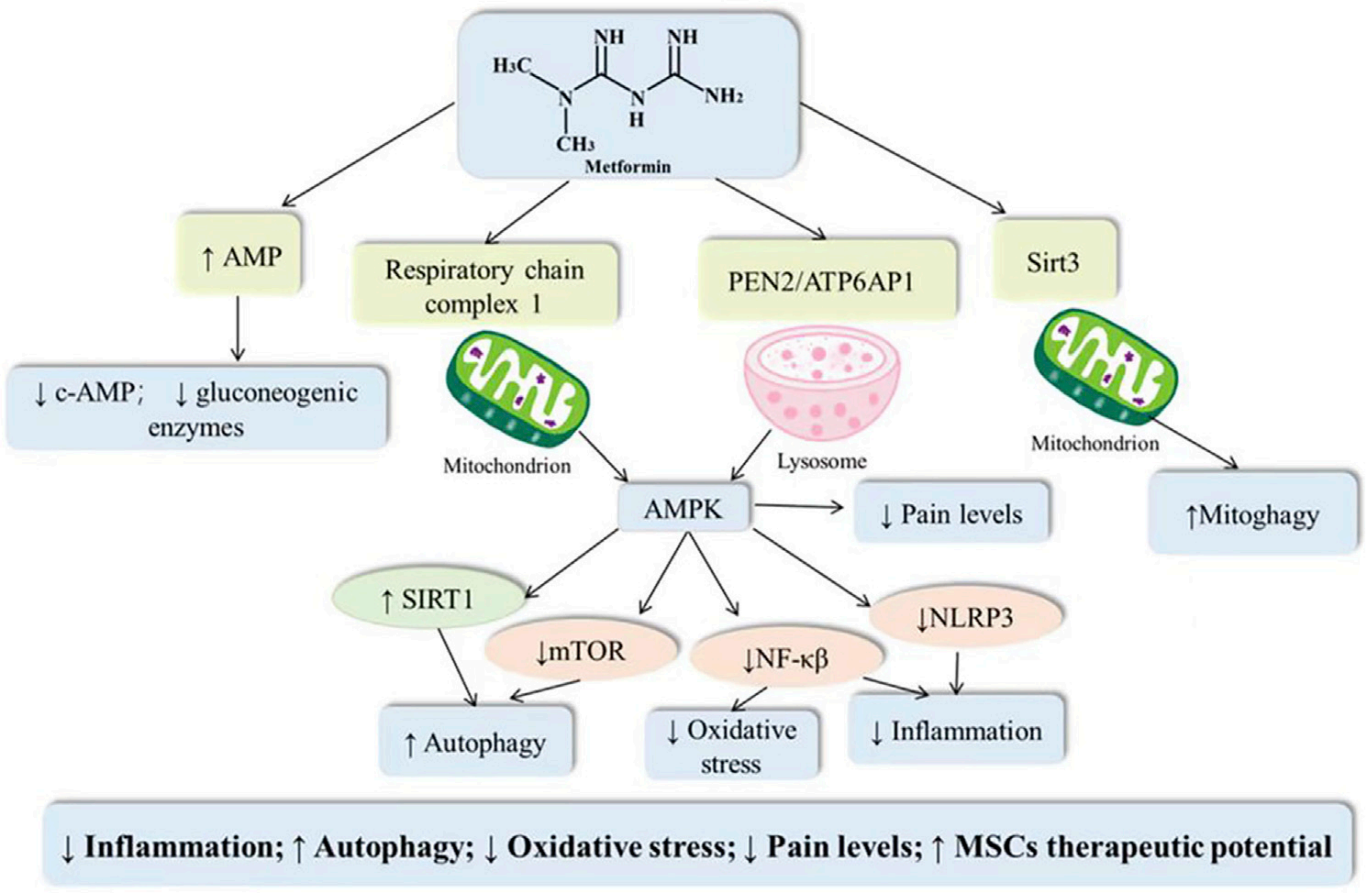
Potential mechanisms of metformin in the treatment of osteoarthritis.

Osteoarthritis is an age-related degenerative joint disease with pathological features including cartilage damage, bone fragmentation, osteophytes, and synovitis. Since cartilage is a non-neurovascular tissue, the target of pharmacological treatment may be the subchondral bone. Abnormal changes in subchondral bone in osteoarthritis are closely associated with both articular pain and articular cartilage degeneration. Long-term chronic inflammatory stimulation, disruption of oxidative and antioxidant balance, and decreased autophagy induce apoptosis of chondrocytes, but these three do not exist independently of each other. Inflammatory storms induce ROS production, and in addition oxidative stress may cause an increased inflammatory response and decreased cellular autophagy, such that the cellular vicious cycle exacerbates the progression of the osteoarthritic disease (Liu-Bryan and Terkeltaub, 2015; Loeser et al., 2016). AMPK, as a central regulator of cellular metabolism, is a key protein involved in the transduction of multiple signaling pathways. Activation of AMPK can not only suppress inflammation, antagonize oxidative stress and regulate autophagy, but also inhibit the transmission of pain signals (Salminen and Kaarniranta, 2012). Metformin, as an AMPK activator, may inhibit chondrocyte apoptosis and improve pain and other related symptoms through multiple mechanisms (As shown in Figure 1).

### Suppression of inflammation

The osteochondral unit is a structural and functional unit composed of articular cartilage, subchondral bone, and calcified cartilage that together play an important part in the disease progression of osteoarthritis (Goldring and Goldring, 2016). It is important to note that osteoarthritis is a total joint disease in which any component of the osteochondral unit, not just the articular cartilage, is affected. The balance between anti-inflammation and pro-inflammation plays an important role in maintaining the normal metabolism of synovial tissue, and patients with osteoarthritis in pathological states often show increased levels of T helper cells (TH17) and reduced numbers of T regulatory cells (Treg) (Moradi et al., 2014; Li et al., 2016). In the cellular microenvironment involved in osteoarthritis, cytokines, chemokines and other components secreted by various cells such as synovial cells, chondrocytes, osteoblasts, osteoclasts, and leukocytes together form a complex inflammatory network (Rogoveanu et al., 2018). Chronic low-grade inflammation mediated by the interaction of the innate immune system and inflammatory mediators has a central function in osteoarthritis (Robinson et al., 2016). The investigators compared the immunological features of early and late osteoarthritis and found a significant increase in monocyte infiltration and expression of inflammatory mediators in early synovial tissue (Benito et al., 2005). Thus, synovitis may exist before the structural changes that appear on imaging. Synovitis may drive the development of osteoarthritis (Sokolove and Lepus, 2013). Elevated levels of inflammatory mediators may promote the production and activation of other enzymes such as matrix metalloproteinases (MMPs) family, a disintegrin and metalloproteinase (ADAM), and a disintegrin and metalloproteinase with thrombospondin motifs (ADAMTS), which can destroy articular cartilage structures by degrading chondrocytes and extracellular matrix (Chevalier et al., 2013; Liu-Bryan and Terkeltaub, 2015; Alonso et al., 2020). Inflammatory mediators (e.g., IL-1β) induce downstream effectors such as NO, phospholipase A2 (PLA2), Prostaglandin E2 (PGE2), and reactive oxygen species (ROS), causing vasodilation, joint pain, and cartilage damage (Wojdasiewicz et al., 2014; Bolduc et al., 2019). Evidence from animal studies suggests that TNF-α and IL-6 promote increased sensitivity of injury receptors to mechanical stimuli in rats (Brenn et al., 2007; Richter et al., 2010). Inhibition of TNF-α can significantly relieve pain, which supports the idea that inflammatory mediators are involved in inducing joint pain (Boettger et al., 2008). In addition, synovial macrophages appear to play a role in bone formation (van Lent et al., 2004). Thus, inflammatory factors are involved in the regulation of multiple pathological features of osteoarthritis.

Metformin, as an AMPK activator, can inhibit inflammatory responses by acting directly or indirectly on several signaling pathways (mTOR/STAT (Kim et al., 2018), sirt1/NF-κβ (Zhang et al., 2020), Dicerase (Luo et al., 2020), and HDAC5/KLF2 (Tian et al., 2019), etc.). Zhang et al. (2020) found that metformin attenuated IL-1β-induced articular cartilage damage by modulating the AMPK/NF-κβ signaling pathway. Similarly, in another MIA-induced osteoarthritis rat model, the expression of IL-1β and IL-17 in synovial tissue was significantly decreased in the metformin-treated group compared with the control group (Na et al., 2021). Given the anti-inflammatory capacity of metformin and the large release of inflammatory mediators in the early stages of osteoarthritis that drive disease progression, we propose that early application of metformin may delay the structural destruction of osteoarthritis.

### Regulation of autophagy

The balance between autophagy and apoptosis plays an important role in the development and progression of osteoarthritis, which is usually characterized by an increase in the level of apoptosis and a decrease in the level of autophagy under pathological conditions (Musumeci et al., 2015; Feng et al., 2020a). Under normal conditions, autophagy can act as an intracellular homeostatic mechanism to degrade damaged proteins and dysfunctional organelles for the cell’s metabolic requirements and renewal (Caramés et al., 2012). In the disease progression of osteoarthritis, autophagy is likely to present as a protective mechanism to maintain chondrocyte homeostasis, but aging and stimulation of the environment to which the cells are exposed (e.g., proinflammatory cytokines) may lead to a decrease in the autophagic clearance capacity of the cells, tipping the balance in favor of chondrocyte apoptosis (Maiuri et al., 2007). Therefore, the activation of autophagy to antagonize apoptosis is of great interest in the treatment of osteoarthritis (Caramés et al., 2012; Chen et al., 2018; Wang et al., 2019a). Treatment with rapamycin was found to significantly reduce the severity of osteoarthritis by a mechanism at least partially attributed to the activation of autophagy (Caramés et al., 2012). Both AMPK and mTOR are important components of autophagy initiation. mTOR is a major negative regulator of autophagy and is commonly overexpressed in human, mouse, and dog osteoarthritic chondrocytes. mTOR upregulation is associated with inhibition of autophagic signaling in articular cartilage, while mTOR-specific ablation may regulate chondrocytes by activating autophagy through the ULK1/AMPK signaling pathway homeostasis (Zhang et al., 2015). AMPK is not only an energy sensor for cells but also activates key kinases that induce autophagy (Herzig and Shaw, 2018). Up-regulated AMPK levels inhibit mTOR and ultimately promote autophagy (Alers et al., 2012). Intra-articular injection of resveratrol has been proven to delay articular chondrocyte degeneration in mice by inducing autophagy through the AMPK/mTOR signaling pathway (Qin et al., 2017). Therefore, it appears that metformin, as an AMPK activator, may also exert chondroprotective effects by regulating autophagy. Metformin treatment was found to dose-dependently enhance AMPK and inhibit mTORC1 in chondrocytes, and an upregulation of autophagy marker expression (LC3) was observed in the treated group (Feng et al., 2020b). Another recent study suggested that the AMPKα2-SIRT1 signaling pathway may mediate the autophagy-enhancing effects of metformin in chondrocytes, in which silencing AMPKα2 but not AMPKα1 decreased the expression of autophagy-associated proteins (Wang et al., 2020). Mitochondrial autophagy is a type of cellular autophagy, which is the process of selective removal of excess, senescent, or damaged mitochondria, and the PINK1/Parkin pathway is the classical method for activating mitochondrial autophagy (Onishi et al., 2021). Mitochondria in osteoarthritic chondrocytes often exhibit oxidative-antioxidative imbalance, disruption of calcium homeostasis, and decreased energy supply due to exposure to numerous damaging factors (Farnaghi et al., 2017; Wei et al., 2017). While the traditional view is that AMPK enhances cellular autophagy and thus promotes the clearance of damaged organelles, new studies suggest that AMPK may directly regulate the initiation of mitochondrial autophagy by a mechanism involving phosphorylation of Ser495 of PINK1 by AMPKα2 (Wang et al., 2018a). Additionally, AMPK activates SIRT3, which is localized downstream in mitochondria, to maintain the integrity and function of mitochondrial DNA (Chen et al., 2018). Metformin can upregulate sirt3 expression levels by mechanisms that might include activation by AMPK and in an AMPK-independent manner. Wang et al. (2019a) found that metformin could exert chondroprotective effects in the treatment of osteoarthritis by activating the sirt3/PINK1/Parkin signaling pathway. Enhancing the autophagy level by enhancing AMPK and thus resetting the balance between autophagy and apoptosis in chondrocytes is a worthwhile direction for future research related to osteoarthritis treatment.

### Resistance to oxidative stress damage

Oxidative stress is usually a state in which the oxidative and antioxidant balance in the body is disrupted and a large accumulation of oxidative products, as well as a reduced capacity of the antioxidant system, can cause tissue damage. Oxidative stress accelerates the progression of osteoarthritis through multiple pathways including the promotion of chondrocyte apoptosis, matrix degradation, and inhibition of autophagy (Portal-Núñez et al., 2016). Low concentrations of ROS in chondrocytes under physiological conditions can participate in cellular signaling and play a role in maintaining metabolic homeostasis. However, excessive accumulation of ROS not only plays a synergistic role in chondrocyte apoptosis induced by inflammatory factors but also activates matrix catabolic factors that can disrupt cartilage integrity. Dysfunction of the antioxidant system is also not negligible, and research evidence suggests that deficiency or dysfunction of antioxidant enzymes such as catalase, SOD, and peroxide oxidoreductase is associated with the development of osteoarthritis (Zhuang et al., 2018; Gu et al., 2019; Nandi et al., 2019). In the pathogenesis of osteoarthritis, NF-κβ (Hu et al., 2019), Nrf-2 (Gao et al., 2019), sirt1 (Feng et al., 2019), and the forkhead box transcription factor (FOXO) (Akasaki et al., 2014) may be involved in the transduction of oxidative stress-related signaling pathways. AMPK as a balancer of cellular metabolic homeostasis can be a target for osteoarthritis therapy by interlinking with the above-mentioned signaling molecules (Jeon, 2016). The antioxidant effect of metformin as an AMPK activator in osteoarthritic models has been demonstrated in a small number of studies (Dawood et al., 2020). Metformin treatment protected against articular cartilage damage secondary to type 2 diabetes in the study by Dawood et al. (2020). This may be due to the inhibition of hyperglycemia, oxidative stress, and inflammation by metformin (Dawood et al., 2020). In another study, SIRT3 expression was decreased in a model of osteoarthritis and correlated with ROS production, and metformin treatment exerted a chondroprotective effect by promoting SIRT3 expression (Wang et al., 2019a).

### Reduction of pain levels

Pain is a common clinical manifestation of osteoarthritis and the main reason for seeking medical attention. The NSAIDs currently used clinically are effective, however, we have to be concerned about the possible adverse effects of long-term use (van Walsem et al., 2015). The development of new pain relief methods to help patients with osteoarthritis is the focus of future research, and AMPK may be a new target for pain treatment, as its activation may play a role in pain management by inhibiting pain-related signaling and reducing nociceptive neuron sensitivity (Price et al., 2016). The transient receptor potential (TRPs) family of ion channels are primary receptors of pain injury and are capable of sensing different stimuli from internal and external contexts to mediate pain signaling. Trpv1 and Trpa1 are widely recognized molecular sensors involved in injury perception and heat sensitivity and play a key role in the propagation of pain and the transition from acute to chronic pain (Spicarova et al., 2014; Choi et al., 2016; Vandewauw et al., 2018; Hao et al., 2019). The analgesic effects of metformin have now been demonstrated in several experiments, including injury models, chronic nerve injury models, chronic inflammation models, and postoperative pain models (Kiałka et al., 2017; Augusto et al., 2019; Li et al., 2020a; Das et al., 2020). An experimental animal study of osteoarthritis revealed that metformin treatment decreased dorsal root ganglion (DRG) pain sensitivity in mice through upregulation of AMPKα1 expression (Li et al., 2020b). Similarly, a study by Li et al. (2020a) suggested that either intra-articular or intragastric application of metformin resulted in improvements in pain-related behaviors. Increased excitability of peripheral sensory neurons in DRG is closely associated with the onset of pain hypersensitivity, and the analgesic effect of metformin is at least partially attributed to the inhibition of membrane-associated TRPA1 expression and TRPA1-mediated calcium inward flow in DRG (Wang et al., 2018b). In addition, in the central nervous system AMPK regulates the neuropeptide calcitonin gene-related peptide (CGRP), a transmitter that initiates and maintains neuropathic pain. Blocking AMPK-CGRP pathway signaling causes increased neuropathic pain sensitivity (Guo et al., 2019). In a sodium iodoacetate (MIA)-induced OA rat model, metformin treatment reduced the expression of the pain-related mediator CGRP in the dorsal root ganglion (DRG) but had no significant effect on the expression of TRPV1 (Na et al., 2021). However, the role of TRPV1 in pain treatment still deserves attention, and studies in a rat model of bone cancer pain have found that metformin treatment can provide strong relief of mechanical abnormal pain by downregulating TRPV1 expression (Qian et al., 2021).

## Beneficial evidence for the application of metformin in osteoarthritis

Osteoarthritis is a disease of the locomotor system closely related to aging and can affect any joint in the body, with joints of the knee, hand, hip, and spine being more common. Under normal circumstances, articular cartilage is located between the contact surfaces of two bones and acts as a cushion to protect the bones during movement. However, articular cartilage has a “lifespan,” and poor exercise habits and poor metabolic environment may accelerate the degeneration of articular cartilage. Epidemiological studies have found a 54% prevalence of osteoarthritis in patients with type 2 diabetes mellitus (T2DM) (Na et al., 2019). High glucose levels not only promote chondrocyte catabolic gene expression and inhibit the differentiation of MSCs() into chondrocytes, but also accelerate the damage of cartilage structures (Rosa et al., 2011; Tsai et al., 2013). Metformin has received a lot of attention from researchers for its role in osteoarthritis models. Our summary of the current research evidence is presented in Table 1.

**TABLE 1:** The therapeutic role of metformin in osteoarthritis.

Type of study	Research subjects	Models	Metformin dosage	Year	References	Effects
*In vivo*	10-week-old male C57 wild-type (WT) mice	DMM	205 mg/kg per day; Before or 2 weeks after surgery	2020	Li et al. (2020b)	Inhibition of cartilage degeneration, osteophyte formation, and pain signaling through activation of AMPKα1
	Male rhesus macaques with 8.5–11.4 years of age	Partial medial meniscectomy (PMM)	51.7 mg/kg per day; 1 month after PMM surgery			
*In vivo*	Eight-week-old C57bl/6 mice	DMM	A stock solution of metformin (1.65 g/ml) was diluted in PBS (1:100) and injected intra-knee every three days for eight weeks	2020	Wang et al. (2020)	Activation of AMPKα2/SIRT1 pathway and thus promotion of chondrocyte autophagy
*In vitro*	Murine chondrocytes	IL-1β (10 ng/ml)-induced chondrocyte injury	Metformin (1 mM) was administrated one hour before the stimulation of IL-1β
*In vitro*	Mouse articular chondrocytes	IL-1β (0, 0.3 1, 3, 10 and 30 ng/ml)	Metformin (0–2 mM)	2019	Wang et al. (2019a)	Activation of SIRT3-mediated PINK1/Parkin-dependent mitophagy
*In vivo*	Six-week-old male Wistar rats	3 mg monosodium iodoacetate (MIA)	Ad-hMSCs were stimulated with metformin (1 mM) for 48 h, and then injected into rats	2019	Park et al. (2019)	Enhancement of immunomodulatory properties and migratory capacity of MSCs, thereby reducing chondrocyte apoptosis and pain
*In vivo*	10-week-old male C57BL/6 mice	DMM	Intragastric group: metformin (200 mg/kg) was given 3 days after the surgery; once daily for 8 weeks; Intraarticular group: metformin (0.1 mmol/kg) was injected into the knee joint cavity 3 days after the surgery; twice a week for 8 weeks	2020	Li et al. (2020a)	Inhibition of catabolism and reduction of pain behavior
*In vitro*	Mouse primary chondrocytes	IL-1β (10 ng/ml)-induced chondrocyte injury	Metformin (1, 10, and 20 mM)
*In vivo*	8-week-old male C57BL/6 mice	DMM	100 mg/kg/d or 200 mg/kg/d metformin received by oral forcible feeding	2020	Feng et al. (2020b)	Regulation of AMPK/mTOR signaling pathway and thus effective mitigation of chondrocyte apoptosis and senescence
*In vitro*	Primary articular chondrocytes	IL-1β (10 ng/ml)-induced chondrocyte injury	Metformin (5 mM/L) for 24 h
*In vivo*	Albino male rats	T2DM induced OA model	The rats started metformin (200 mg/kg body weight) treatment 14 days before diabetic induction and continued on metformin until the end of the experiment at week 12	2020	Dawood et al. (2020)	Anti-inflammation and inhibition of oxidative stress, thereby preventing and alleviating severe damage to the ultrastructure of articular cartilage
*In vitro*	The murine ATDC5 chondrocyte cell line	L-1β (10 ng/ml)-induced chondrocyte injury	Metformin (1 mM) for 24 h	2020	Zhang et al. (2020)	Regulation of the AMPK/NF-κβ pathway thereby promoting extracellular matrix (ECM) metabolic homeostasis, suppressing inflammatory responses, and promoting cell proliferation
*In vivo*	8-week-old C57BL/6 male mice	DMM	200 mg/kg for 4 or 8 weeks	2022	Yan et al. (2022)	Inhibition of NLRP3 inflammasome activation to reduce cartilage degradation, subchondral bone remodeling, and chondrocyte scorching
*In vivo*	Seven-week-old male Wistar rats	MIA	Metformin (100 mg/kg) was orally administered using an oral gavage needle for 14 days	2021	Na et al. (2021)	Modulation of pain mediators and autophagy-lysosome pathways to show analgesic properties and chondroprotection
*In vitro*	Human articular chondrocytes	Patients undergoing replacement arthroplasty or joint replacement surgery	Metformin (1 mM) for 24 h
*In vitro*	Human articular chondrocytes	Female patients with end-stage knee OA	1 mM metformin and incubated for 48 h	2020	Schadler et al. (2021)	Reduced expression of catabolic genes
Prospective cohort study	Human	In obese people with knee osteoarthritis	Administration of metformin	2019	Wang et al. (2019b)	Inhibition of cartilage volume loss and reduction in risk of total joint replacement
Retrospective, matched-cohort study	Human	In osteoarthritis patients with T2DM	combination of COX-2 inhibitors and metformin therapy	2018	Lu et al. (2018)	Combination therapy resulted in lower rates of joint replacement surgery
Randomized double-blind study	Human	In patients who have symptomatic and radiologic evidence of painful OA of the knee	metformin (1000 mg/day) + meloxicam (15 mg/day)	2014	Mohammed et al. (2014)	As an adjuvant to better promote the analgesic and anti-inflammatory effects of NSAIDs
Electronic health record cohort study	Human	In patients with T2DM	Administration of metformin	2017	Barnett et al. (2017)	No significant association between metformin treatment and the development of osteoarthritis in diabetic patients

### Animal

In a model of osteoarthritis secondary to diabetes, metformin prevented and delayed the progression of osteoarthritis by inhibiting inflammation and oxidative stress damage (Dawood et al., 2020). One question that deserves to be explored is whether the protective effect of metformin on osteoarthritis is limited to diabetic individuals. Metformin is also protective in the medial meniscus instability (DMM) or monosodium iodoacetate (MIA)-induced osteoarthritis model in mice. Metformin treatment delays chondrocyte senescence and thus contributes to the treatment of osteoarthritis (Feng et al., 2020b). Similarly, a study by Wang et al. (2020) found that joint cavity injection of metformin inhibited chondrocyte apoptosis and extracellular matrix degradation through activation of autophagy. Additionally, the inhibition of NLRP3 inflammasome activation, reduction of cartilage degradation, and subchondral bone remodeling was also demonstrated in the study (Yan et al., 2022). In a DMM-induced osteoarthritis model, metformin inhibited articular cartilage degeneration, synovial tissue proliferation, osteophyte formation, and pain-related signaling through upregulation of AMPKα1 expression. The accelerated disease progression in AMPKα1 knockout mice suggested that AMPKα1 may have a protective role, and metformin amplified this effect. Moreover, the investigators explored the effects of metformin in non-human primates, rhesus monkeys, and the higher cartilage thickness and prolonged standing and walking time supported metformin as a disease-modifying agent for OA (Li et al., 2020b). The study investigated whether the protective effects of metformin differed depending on the mode of administration and found that metformin was chondroprotective whether applied intra-gastrically or intra-articularly (respectively, compared to the control group) (Li et al., 2020a). However, there are no studies comparing which administration method is more effective. The expression levels of cartilage matrix-degrading enzymes (MMP-13 and MMP-3) were inversely correlated with metformin concentration compared to the control group, and the degree of cartilage matrix degradation in OA improved significantly with the increasing number of metformin applications (Feng et al., 2020b). In a model of MIA-induced osteoarthritis, metformin exerted chondroprotective effects by modulating pain mediators and promoting autophagy, and the study also found that the combination of metformin and celecoxib was more effective (Na et al., 2021). Besides, *in vitro* culture of mouse chondrocytes revealed that metformin treatment (1 mM) not only activated the AMPK/NF-κβ signaling pathway to promote cell-matrix metabolic homeostasis, inhibit the inflammatory response and chondrocyte apoptosis (Zhang et al., 2020), but also regulated AMPKα2/sirt1 signaling pathway to activate autophagy (Wang et al., 2020). In another study metformin (0–2 mM) upregulated mitochondrial autophagy by activating SIRT3 (Wang et al., 2019a). Among the common current treatments for osteoarthritis long-term oral analgesics may exacerbate gastric mucosal damage, the effects of intra-articular hyaluronic acid injections are uncertain (Rosen et al., 2016), and late joint replacement surgery may pose other physical challenges for older adults (Snell et al., 2021). The repair of the articular cartilage remains an unresolved challenge, and stem cell therapy is gaining attention as an emerging tool for researchers. However, current data from basic and clinical trials have found limited efficacy of intra-articular MSC (Mesenchymal Stem Cell) injections for the treatment of osteoarthritis (Davatchi et al., 2011; Farrell et al., 2016). Interestingly, Park et al. (2019) found that metformin-treated MSCs effectively inhibited cartilage degeneration and exerted analgesic properties, which may be attributed to the enhanced immunomodulatory properties and migration ability of MSCs by metformin. Most of the above studies were performed *in vivo* or *in vitro* in mice, and the results of the animal studies suggest that the chondroprotective effect of metformin is not limited to osteoarthritis secondary to diabetes, but is also effective in models of DMM or MIA-induced osteoarthritis. The mode of administration encompassed both intragastric and intra-articular cavities and the therapeutic effect was correlated with concentration and number of applications. In conclusion, the evidence from these animal studies supports metformin as a promising drug for the treatment of osteoarthritis.

### Human

The protective potential of metformin in osteoarthritis is also supported by results from human clinical studies. Data from an *in vitro* culture study of human cells found that metformin treatment suppressed the expression of catabolic-related genes and the results of this study supported that metformin still had a regulatory effect on chondrocytes in patients with advanced osteoarthritis (Schadler et al., 2021). Similarly, another study cultured articular chondrocytes from patients undergoing arthroplasty or phalangeal surgery and found that metformin promoted the expression of autophagic lysosomal markers (Na et al., 2021). A prospective cohort study enrolling 818 obese patients with knee osteoarthritis found that metformin use was associated with reduced knee cartilage volume over 4 years and a reduced risk of total knee replacement over 6 years (Wang et al., 2019b). Another study from Taiwanese patients with type 2 diabetes found that combined treatment with metformin and COX-2 inhibitors was able to reduce the rate of joint replacement surgery by 25% over 10 years compared to COX-2 inhibitors alone. Metformin synergistically inhibited the release of pro-inflammatory cytokines (Lu et al., 2018). Moreover, a randomized, double-blind clinical trial identified the ability of adjuvant metformin treatment to increase the analgesic and anti-inflammatory effects of meloxicam (Mohammed et al., 2014). However, not all studies have reached consistent conclusions, and Barnett et al. suggested that metformin treatment was not significantly associated with the development of osteoarthritis in diabetic patients. Possible influencing factors are the incomplete consideration of confounding factors and the lack of accuracy of diagnostic methods for OA. In addition, focusing only on whether metformin is applied in diabetic patients without investigating the specific dose and duration of use may also have an impact on the conclusions (Barnett et al., 2017). Current clinical studies from humans are mostly limited to diabetic patients, probably because the effects of metformin are still limited to lowering blood glucose, and it is difficult to evaluate the effects of metformin in healthy populations for the prevention of osteoarthritis. In recent years, metformin has become an anti-cancer and anti-aging drug, and the US Food and Drug Administration (FDA) has approved the first clinical trial study of metformin in non-diabetic individuals (Targeting Ageing with Metformin, TAME) in 2015, but the results of the study have not been updated yet (Barzilai et al., 2016). More high-quality studies exploring the relationship between metformin and osteoarthritis are needed in the future.

## Potential side effects of metformin

Digestive disorders (such as nausea, vomiting, diarrhea, upset stomach, etc.) are the most common side effects of metformin, but these are often transient and mild (Bouchoucha et al., 2011). In addition, the effect of metformin on gastrointestinal function may lead to vitamin B12 deficiency due to poor digestion and absorption of vitamin B12. A cross-sectional study found a significant correlation between metformin at ≥1,500 mg/d and vitamin B12 deficiency (Kim et al., 2019). Lower vitamin B12 levels may be associated with neuropathy, microangiopathy, and cardiovascular disease (Lin et al., 2012; Vinik et al., 2013; Fotiou et al., 2014), but the current view is that VitB12 deficiency triggered by metformin is usually not severe. Metformin-associated lactic acidosis is rare but dangerous, with a mortality rate of up to 50%, and can be triggered by excessive drug doses and abnormal liver and kidney function (DeFronzo et al., 2016). Therefore, the dosage of metformin should be reduced as appropriate in people with renal impairment. Since its approval by the US Food and Drug Administration (FDA) in 1995, the maximum daily dose of metformin for the treatment of type 2 diabetes should not exceed 2,000 mg. Considering the side effects of metformin and the possible individual differences, metformin treatment should take into account the age, physical condition, liver and kidney function, and gastrointestinal function of the individual.

## Conclusion and perspectives

In summary, evidence for chondroprotection by metformin has been reported from animal and clinical studies, where animal studies were not limited to osteoarthritis models secondary to diabetes, but metformin also showed protective effects in DMM and MIA-induced osteoarthritis. This may suggest that metformin exerts its chondroprotective benefits through other pathways than just lowering blood glucose. It is important to note that osteoarthritis is a disease in which multiple factors come together. People with diabetes or metabolic abnormalities are at increased risk of developing osteoarthritis, but healthy individuals also experience degeneration of joint cartilage with age. The search for effective treatments is of clinical importance. Our review identified metformin as a promising drug for the treatment of osteoarthritis by reviewing recent studies, which could play a role in suppressing inflammation, regulating autophagy, antagonizing oxidative stress, and reducing pain levels by activating AMPK and thus regulating the transduction of downstream signaling molecules. However, generally speaking, the data available in this area are scarce and most of the studies are on mice. More high-quality studies are needed in the future to further explore the possibilities of metformin in the treatment of osteoarthritis. Metformin has a long way to go in the clinical management of osteoarthritis, but it is worth acknowledging that it is indeed a promising drug.

## References

[B1] Agius L. Ford B. E. Chachra S. S. (2020). The metformin mechanism on gluconeogenesis and AMPK activation: The metabolite perspective. Int. J. Mol. Sci. 21 (9), E3240. 10.3390/ijms21093240 32375255 PMC7247334

[B2] Akasaki Y. Alvarez-Garcia O. Saito M. Caramés B. Iwamoto Y. Lotz M. K. (2014). FoxO transcription factors support oxidative stress resistance in human chondrocytes. Arthritis Rheumatol. 66 (12), 3349–3358. 10.1002/art.38868 25186470 PMC4254812

[B3] Alers S. Löffler A. S. Wesselborg S. Stork B. (2012). Role of AMPK-mTOR-ulk1/2 in the regulation of autophagy: Cross talk, shortcuts, and feedbacks. Mol. Cell. Biol. 32 (1), 2–11. 10.1128/MCB.06159-11 22025673 PMC3255710

[B4] Alonso B. Bravo B. Mediavilla L. Gortazar A. R. Forriol F. Vaquero J. (2020). Osteoarthritis-related biomarkers profile in chronic anterior cruciate ligament injured knee. Knee 27 (1), 51–60. 10.1016/j.knee.2019.12.007 31926672

[B5] Alshawi A. Agius L. (2019). Low metformin causes a more oxidized mitochondrial NADH/NAD redox state in hepatocytes and inhibits gluconeogenesis by a redox-independent mechanism. J. Biol. Chem. 294 (8), 2839–2853. 10.1074/jbc.RA118.006670 30591586 PMC6393620

[B6] Alušík Š. Paluch Z. (2015). Metformin: The past, presence, and future. Minerva Med. 106 (4), 233–238. 25532538

[B7] Asiedu M. N. Dussor G. Price T. J. (2016). Targeting AMPK for the alleviation of pathological pain. Exp. Suppl. 107, 257–285. 10.1007/978-3-319-43589-3_11 27812984 PMC5715474

[B8] Augusto P. Braga A. V. Rodrigues F. F. Morais M. I. Dutra M. M. G. B. Batista C. R. A. (2019). Metformin antinociceptive effect in models of nociceptive and neuropathic pain is partially mediated by activation of opioidergic mechanisms. Eur. J. Pharmacol. 858, 172497. 10.1016/j.ejphar.2019.172497 31238066

[B9] Barnett L. A. Jordan K. P. Edwards J. J. van der Windt D. A. (2017). Does metformin protect against osteoarthritis? An electronic health record cohort study. Prim. Health Care Res. Dev. 18 (6), 623–628. 10.1017/S1463423617000287 28539134

[B10] Barzilai N. Crandall J. P. Kritchevsky S. B. Espeland M. A. (2016). Metformin as a tool to target aging. Cell Metab. 23 (6), 1060–1065. 10.1016/j.cmet.2016.05.011 27304507 PMC5943638

[B11] Benito M. J. Veale D. J. FitzGerald O. van den Berg W. B. Bresnihan B. (2005). Synovial tissue inflammation in early and late osteoarthritis. Ann. Rheum. Dis. 64 (9), 1263–1267. 10.1136/ard.2004.025270 15731292 PMC1755629

[B12] Boettger M. K. Hensellek S. Richter F. Gajda M. Stockigt R. von Banchet G. S. (2008). Antinociceptive effects of tumor necrosis factor alpha neutralization in a rat model of antigen-induced arthritis: Evidence of a neuronal target. Arthritis Rheum. 58 (8), 2368–2378. 10.1002/art.23608 18668541

[B13] Bolduc J. A. Collins J. A. Loeser R. F. (2019). Reactive oxygen species, aging and articular cartilage homeostasis. Free Radic. Biol. Med. 132, 73–82. 10.1016/j.freeradbiomed.2018.08.038 30176344 PMC6342625

[B14] Bouchoucha M. Uzzan B. Cohen R. (2011). Metformin and digestive disorders. Diabetes Metab. 37 (2), 90–96. 10.1016/j.diabet.2010.11.002 21236717

[B15] Brenn D. Richter F. Schaible H. G. (2007). Sensitization of unmyelinated sensory fibers of the joint nerve to mechanical stimuli by interleukin-6 in the rat: An inflammatory mechanism of joint pain. Arthritis Rheum. 56 (1), 351–359. 10.1002/art.22282 17195239

[B16] Caramés B. Hasegawa A. Taniguchi N. Miyaki S. Blanco F. J. Lotz M. (2012). Autophagy activation by rapamycin reduces severity of experimental osteoarthritis. Ann. Rheum. Dis. 71 (4), 575–581. 10.1136/annrheumdis-2011-200557 22084394 PMC3294168

[B17] Chen L. Y. Wang Y. Terkeltaub R. Liu-Bryan R. (2018). Activation of AMPK-SIRT3 signaling is chondroprotective by preserving mitochondrial DNA integrity and function. Osteoarthr. Cartil. 26 (11), 1539–1550. 10.1016/j.joca.2018.07.004 PMC6202232 30031925

[B18] Chevalier X. Eymard F. Richette P. (2013). Biologic agents in osteoarthritis: Hopes and disappointments. Nat. Rev. Rheumatol. 9 (7), 400–410. 10.1038/nrrheum.2013.44 23545735

[B19] Choi S. I. Lim J. Y. Yoo S. Kim H. Hwang S. W. (2016). Emerging role of spinal cord TRPV1 in pain exacerbation. Neural Plast. 2016, 5954890. 10.1155/2016/5954890 26885404 PMC4738952

[B20] Das V. Kroin J. S. Moric M. McCarthy R. J. Buvanendran A. (2020). Early treatment with metformin in a mice model of complex regional pain syndrome reduces pain and edema. Anesth. Analg. 130 (2), 525–534. 10.1213/ANE.0000000000004057 30801357

[B21] Davatchi F. Abdollahi B. S. Mohyeddin M. Shahram F. Nikbin B. (2011). Mesenchymal stem cell therapy for knee osteoarthritis. Preliminary report of four patients. Int. J. Rheum. Dis. 14 (2), 211–215. 10.1111/j.1756-185X.2011.01599.x 21518322

[B22] Dawood A. F. Alzamil N. Ebrahim H. A. Abdel Kader D. H. Kamar S. S. Haidara M. A. (2020). Metformin pretreatment suppresses alterations to the articular cartilage ultrastructure and knee joint tissue damage secondary to type 2 diabetes mellitus in rats. Ultrastruct. Pathol. 44 (3), 273–282. 10.1080/01913123.2020.1762815 32404018

[B23] DeFronzo R. Fleming G. A. Chen K. Bicsak T. A. (2016). Metformin-associated lactic acidosis: Current perspectives on causes and risk. Metabolism. 65 (2), 20–29. 10.1016/j.metabol.2015.10.014 26773926

[B24] Farnaghi S. Prasadam I. Cai G. Friis T. Du Z. Crawford R. (2017). Protective effects of mitochondria-targeted antioxidants and statins on cholesterol-induced osteoarthritis. FASEB J. 31 (1), 356–367. 10.1096/fj.201600600R 27737897

[B25] Farrell E. Fahy N. Ryan A. E. Flatharta C. O. O'Flynn L. Ritter T. (2016). vIL-10-overexpressing human MSCs modulate naïve and activated T lymphocytes following induction of collagenase-induced osteoarthritis. Stem Cell Res. Ther. 7 (1), 74. 10.1186/s13287-016-0331-2 27194025 PMC4870800

[B26] Feng K. Chen Z. Pengcheng L. Zhang S. Wang X. (2019). Quercetin attenuates oxidative stress-induced apoptosis via SIRT1/AMPK-mediated inhibition of ER stress in rat chondrocytes and prevents the progression of osteoarthritis in a rat model. J. Cell. Physiol. 234 (10), 18192–18205. 10.1002/jcp.28452 30854676

[B27] Feng L. Feng C. Wang C. X. Xu D. Y. Chen J. J. Huang J. F. (2020). Circulating microRNA let-7e is decreased in knee osteoarthritis, accompanied by elevated apoptosis and reduced autophagy. Int. J. Mol. Med. 45 (5), 1464–1476. 10.3892/ijmm.2020.4534 32323821 PMC7138275

[B28] Feng X. Pan J. Li J. Zeng C. Qi W. Shao Y. (2020). Metformin attenuates cartilage degeneration in an experimental osteoarthritis model by regulating AMPK/mTOR. Aging (Albany NY) 12 (2), 1087–1103. 10.18632/aging.102635 31945013 PMC7053618

[B29] Foretz M. Guigas B. Viollet B. (2019). Understanding the glucoregulatory mechanisms of metformin in type 2 diabetes mellitus. Nat. Rev. Endocrinol. 15 (10), 569–589. 10.1038/s41574-019-0242-2 31439934

[B30] Foretz M. Hébrard S. Leclerc J. Zarrinpashneh E. Soty M. Mithieux G. (2010). Metformin inhibits hepatic gluconeogenesis in mice independently of the LKB1/AMPK pathway via a decrease in hepatic energy state. J. Clin. Invest. 120 (7), 2355–2369. 10.1172/JCI40671 20577053 PMC2898585

[B31] Fotiou P. Raptis A. Apergis G. Dimitriadis G. Vergados I. Theodossiadis P. (2014). Vitamin status as a determinant of serum homocysteine concentration in type 2 diabetic retinopathy. J. Diabetes Res. 2014, 807209. 10.1155/2014/807209 25006590 PMC4071945

[B32] Fyffe F. A. Hawley S. A. Gray A. Hardie D. G. (2018). Cell-free assays to measure effects of regulatory ligands on AMPK. Methods Mol. Biol. 1732, 69–86. 10.1007/978-1-4939-7598-3_5 29480469

[B33] Gao X. Jiang S. Du Z. Ke A. Liang Q. Li X. (2019). KLF2 protects against osteoarthritis by repressing oxidative response through activation of Nrf2/ARE signaling *in vitro* and *in vivo* . Oxid. Med. Cell. Longev. 2019, 8564681. 10.1155/2019/8564681 31827706 PMC6885785

[B34] Goldring S. R. Goldring M. B. (2016). Changes in the osteochondral unit during osteoarthritis: Structure, function and cartilage-bone crosstalk. Nat. Rev. Rheumatol. 12 (11), 632–644. 10.1038/nrrheum.2016.148 27652499

[B35] Gu Y. Ren K. Jiang C. Wang L. Yao Q. (2019). Regulation of cartilage damage caused by lack of Klotho with thioredoxin/peroxiredoxin (Trx/Prx) system and succedent NLRP3 activation in osteoarthritis mice. Am. J. Transl. Res. 11 (12), 7338–7350. 31934282 PMC6943451

[B36] Guo X. Tao X. Tong Q. Li T. Dong D. Zhang B. (2019). Impaired AMPK-CGRP signaling in the central nervous system contributes to enhanced neuropathic pain in high-fat diet-induced obese rats, with or without nerve injury. Mol. Med. Rep. 20 (2), 1279–1287. 10.3892/mmr.2019.10368 31173269 PMC6625401

[B37] Hao Y. Luo X. Ba X. Wang J. Zhou S. Yang S. (2019). Huachansu suppresses TRPV1 up-regulation and spinal astrocyte activation to prevent oxaliplatin-induced peripheral neuropathic pain in rats. Gene 680, 43–50. 10.1016/j.gene.2018.09.035 30244138

[B38] Hawley S. A. Ross F. A. Chevtzoff C. Green K. A. Evans A. Fogarty S. (2010). Use of cells expressing gamma subunit variants to identify diverse mechanisms of AMPK activation. Cell Metab. 11 (6), 554–565. 10.1016/j.cmet.2010.04.001 20519126 PMC2935965

[B39] He L. (2020). Metformin and systemic metabolism. Trends Pharmacol. Sci. 41 (11), 868–881. 10.1016/j.tips.2020.09.001 32994049 PMC7572679

[B40] He L. Wondisford F. E. (2015). Metformin action: Concentrations matter. Cell Metab. 21 (2), 159–162. 10.1016/j.cmet.2015.01.003 25651170

[B41] Herzig S. Shaw R. J. (2018). Ampk: Guardian of metabolism and mitochondrial homeostasis. Nat. Rev. Mol. Cell Biol. 19 (2), 121–135. 10.1038/nrm.2017.95 28974774 PMC5780224

[B42] Hu N. Gong X. Yin S. Li Q. Chen H. Li Y. (2019). Saxagliptin suppresses degradation of type II collagen and aggrecan in primary human chondrocytes: A therapeutic implication in osteoarthritis. Artif. Cells Nanomed. Biotechnol. 47 (1), 3239–3245. 10.1080/21691401.2019.1647223 31364869

[B43] Hunter D. J. Bierma-Zeinstra S. (2019). Osteoarthritis. Lancet 393 (10182), 1745–1759. 10.1016/S0140-6736(19)30417-9 31034380

[B44] Jeon S. M. (2016). Regulation and function of AMPK in physiology and diseases. Exp. Mol. Med. 48 (7), e245. 10.1038/emm.2016.81 27416781 PMC4973318

[B45] Keller U. Berger W. (1983). Oral antidiabetic agents: Recent aspects. Schweiz. Med. wochenschr. 113 (17), 645–650. 6191388

[B46] Kiałka M. Doroszewska K. Janeczko M. Milewicz T. (2017). Metformin–new potential medicine in pain treatment? Przegl. Lek. 74 (2), 81–83. 29694764

[B47] Kim E. K. Lee S. H. Lee S. Y. Kim J. K. Jhun J. Y. Na H. S. (2018). Metformin ameliorates experimental-obesity-associated autoimmune arthritis by inducing FGF21 expression and Brown adipocyte differentiation. Exp. Mol. Med. 50 (1), e432. 10.1038/emm.2017.245 29371695 PMC5799802

[B48] Kim J. Ahn C. W. Fang S. Lee H. S. Park J. S. (2019). Association between metformin dose and vitamin B12 deficiency in patients with type 2 diabetes. Med. Baltim. 98 (46), e17918. 10.1097/MD.0000000000017918 PMC6867725 31725641

[B49] Li H. Ding X. Terkeltaub R. Lin H. Zhang Y. Zhou B. (2020). Exploration of metformin as novel therapy for osteoarthritis: Preventing cartilage degeneration and reducing pain behavior. Arthritis Res. Ther. 22 (1), 34. 10.1186/s13075-020-2129-y 32087740 PMC7036179

[B50] Li J. Zhang B. Liu W. X. Lu K. Pan H. Wang T. (2020). Metformin limits osteoarthritis development and progression through activation of AMPK signalling. Ann. Rheum. Dis. 79 (5), 635–645. 10.1136/annrheumdis-2019-216713 32156705 PMC7213329

[B51] Li S. Wan J. Anderson W. Sun H. Zhang H. Peng X. (2016). Downregulation of IL-10 secretion by Treg cells in osteoarthritis is associated with a reduction in Tim-3 expression. Biomed. Pharmacother. 79, 159–165. 10.1016/j.biopha.2016.01.036 27044824

[B52] Lin X. Lu D. Gao Y. Tao S. Yang X. Feng J. (2012). Genome-wide association study identifies novel loci associated with serum level of vitamin B12 in Chinese men. Hum. Mol. Genet. 21 (11), 2610–2617. 10.1093/hmg/dds062 22367966

[B53] Liu-Bryan R. Terkeltaub R. (2015). Emerging regulators of the inflammatory process in osteoarthritis. Nat. Rev. Rheumatol. 11 (1), 35–44. 10.1038/nrrheum.2014.162 25266449 PMC4374654

[B54] Loeser R. F. Collins J. A. Diekman B. O. (2016). Ageing and the pathogenesis of osteoarthritis. Nat. Rev. Rheumatol. 12 (7), 412–420. 10.1038/nrrheum.2016.65 27192932 PMC4938009

[B55] Lu C. H. Chung C. H. Lee C. H. Hsieh C. H. Hung Y. J. Lin F. H. (2018). Combination COX-2 inhibitor and metformin attenuate rate of joint replacement in osteoarthritis with diabetes: A nationwide, retrospective, matched-cohort study in taiwan. PLoS One 13 (1), e0191242. 10.1371/journal.pone.0191242 29385156 PMC5791980

[B56] Luo X. Hu R. Zheng Y. Liu S. Zhou Z. (2020). Metformin shows anti-inflammatory effects in murine macrophages through Dicer/microribonucleic acid-34a-5p and microribonucleic acid-125b-5p. J. Diabetes Investig. 11 (1), 101–109. 10.1111/jdi.13074 PMC6944836 31102492

[B57] Lv Z. Guo Y. (2020). Metformin and its benefits for various diseases. Front. Endocrinol. 11, 191. 10.3389/fendo.2020.00191 PMC7212476 32425881

[B58] Ma T. Tian X. Zhang B. Li M. Wang Y. Yang C. (2022). Low-dose metformin targets the lysosomal AMPK pathway through PEN2. Nature 603 (7899), 159–165. 10.1038/s41586-022-04431-8 35197629 PMC8891018

[B59] Maiuri M. C. Zalckvar E. Kimchi A. Kroemer G. (2007). Self-eating and self-killing: Crosstalk between autophagy and apoptosis. Nat. Rev. Mol. Cell Biol. 8 (9), 741–752. 10.1038/nrm2239 17717517

[B60] Miller R. A. Chu Q. Xie J. Foretz M. Viollet B. Birnbaum M. J. (2013). Biguanides suppress hepatic glucagon signalling by decreasing production of cyclic AMP. Nature 494 (7436), 256–260. 10.1038/nature11808 23292513 PMC3573218

[B61] Mohammed M. M. Al-Shamma K. J. Jassim N. A. (2014). Evaluation of the clinical use of metformin or pioglitazone in combination with meloxicam in patients with knee osteoarthritis; using knee injury and osteoarthritis outcome score. Iraqi J. Pharm. Sci. (P-ISSN 1683-3597, E-ISSN 2521-3512) 23 (2), 13–23.

[B62] Moradi B. Schnatzer P. Hagmann S. Rosshirt N. Gotterbarm T. Kretzer J. P. (2014). CD4⁺CD25⁺/highCD127low/⁻ regulatory T cells are enriched in rheumatoid arthritis and osteoarthritis joints--analysis of frequency and phenotype in synovial membrane, synovial fluid and peripheral blood. Arthritis Res. Ther. 16 (2), R97. 10.1186/ar4545 24742142 PMC4060198

[B63] Musumeci G. Castrogiovanni P. Trovato F. M. Weinberg A. M. Al-Wasiyah M. K. Alqahtani M. H. (2015). Biomarkers of chondrocyte apoptosis and autophagy in osteoarthritis. Int. J. Mol. Sci. 16 (9), 20560–20575. 10.3390/ijms160920560 26334269 PMC4613218

[B64] Na A. Jansky L. Gugala Z. (2019). Clinical characteristics of patients with type 2 diabetes mellitus receiving a primary total knee or hip arthroplasty. J. Diabetes Res. 2019, 9459206. 10.1155/2019/9459206 31828171 PMC6885807

[B65] Na H. S. Kwon J. Y. Lee S. Y. Lee S. H. Lee A. R. Woo J. S. (2021). Metformin attenuates monosodium-iodoacetate-induced osteoarthritis via regulation of pain mediators and the autophagy-lysosomal pathway. Cells 10 (3), 681. 10.3390/cells10030681 33808727 PMC8003384

[B66] Nandi A. Yan L. J. Jana C. K. Das N. (2019). Role of catalase in oxidative stress- and age-associated degenerative diseases. Oxid. Med. Cell. Longev. 2019, 9613090. 10.1155/2019/9613090 31827713 PMC6885225

[B67] Onishi M. Yamano K. Sato M. Matsuda N. Okamoto K. (2021). Molecular mechanisms and physiological functions of mitophagy. EMBO J. 40 (3), e104705. 10.15252/embj.2020104705 33438778 PMC7849173

[B68] Palazzo C. Nguyen C. Lefevre-Colau M. M. Rannou F. Poiraudeau S. (2016). Risk factors and burden of osteoarthritis. Ann. Phys. Rehabil. Med. 59 (3), 134–138. 10.1016/j.rehab.2016.01.006 26904959

[B69] Park M. J. Moon S. J. Baek J. A. Lee E. J. Jung K. A. Kim E. K. (2019). Metformin augments anti-inflammatory and chondroprotective properties of mesenchymal stem cells in experimental osteoarthritis. J. Immunol. 203 (1), 127–136. 10.4049/jimmunol.1800006 31142603

[B70] Petursson F. Husa M. June R. Lotz M. Terkeltaub R. Liu-Bryan R. (2013). Linked decreases in liver kinase B1 and AMP-activated protein kinase activity modulate matrix catabolic responses to biomechanical injury in chondrocytes. Arthritis Res. Ther. 15 (4), R77. 10.1186/ar4254 23883619 PMC3979085

[B71] Portal-Núñez S. Esbrit P. Alcaraz M. J. Largo R. (2016). Oxidative stress, autophagy, epigenetic changes and regulation by miRNAs as potential therapeutic targets in osteoarthritis. Biochem. Pharmacol. 108, 1–10. 10.1016/j.bcp.2015.12.012 26711691

[B72] Price T. J. Das V. Dussor G. (2016). Adenosine monophosphate-activated protein kinase (AMPK) activators for the prevention, treatment and potential reversal of pathological pain. Curr. Drug Targets 17 (8), 908–920. 10.2174/1389450116666151102095046 26521775 PMC4852160

[B73] Qian H. Y. Zhou F. Wu R. Cao X. J. Zhu T. Yuan H. D. (2021). Metformin attenuates bone cancer pain by reducing TRPV1 and ASIC3 expression. Front. Pharmacol. 12, 713944. 10.3389/fphar.2021.713944 34421611 PMC8371459

[B74] Qin N. Wei L. Li W. Yang W. Cai L. Qian Z. (2017). Local intra-articular injection of resveratrol delays cartilage degeneration in C57BL/6 mice by inducing autophagy via AMPK/mTOR pathway. J. Pharmacol. Sci. 134 (3), 166–174. 10.1016/j.jphs.2017.06.002 28669597

[B75] Rena G. Hardie D. G. Pearson E. R. (2017). The mechanisms of action of metformin. Diabetologia 60 (9), 1577–1585. 10.1007/s00125-017-4342-z 28776086 PMC5552828

[B76] Richter F. Natura G. Löser S. Schmidt K. Viisanen H. Schaible H. G. (2010). Tumor necrosis factor causes persistent sensitization of joint nociceptors to mechanical stimuli in rats. Arthritis Rheum. 62 (12), 3806–3814. 10.1002/art.27715 20722011

[B77] Robinson W. H. Lepus C. M. Wang Q. Raghu H. Mao R. Lindstrom T. M. (2016). Low-grade inflammation as a key mediator of the pathogenesis of osteoarthritis. Nat. Rev. Rheumatol. 12 (10), 580–592. 10.1038/nrrheum.2016.136 27539668 PMC5500215

[B78] Rogoveanu O. C. Calina D. Cucu M. G. Burada F. Docea A. O. Sosoi S. (2018). Association of cytokine gene polymorphisms with osteoarthritis susceptibility. Exp. Ther. Med. 16 (3), 2659–2664. 10.3892/etm.2018.6477 30186498 PMC6122495

[B79] Romero R. Erez O. Hüttemann M. Maymon E. Panaitescu B. Conde-Agudelo A. (2017). Metformin, the aspirin of the 21st century: its role in gestational diabetes mellitus, prevention of preeclampsia and cancer, and the promotion of longevity. Am. J. Obstet. Gynecol. 217 (3), 282–302. 10.1016/j.ajog.2017.06.003 28619690 PMC6084482

[B80] Rosa S. C. Rufino A. T. Judas F. M. Tenreiro C. M. Lopes M. C. Mendes A. F. (2011). Role of glucose as a modulator of anabolic and catabolic gene expression in normal and osteoarthritic human chondrocytes. J. Cell. Biochem. 112 (10), 2813–2824. 10.1002/jcb.23196 21608018

[B81] Rosen J. Avram V. Fierlinger A. Niazi F. Sancheti P. Bedi A. (2016). Clinicians' perspectives on the use of intra-articular hyaluronic acid as a treatment for knee osteoarthritis: A north American, multidisciplinary survey. Clin. Med. Insights. Arthritis Musculoskelet. Disord. 9, 21–27. 10.4137/CMAMD.S34496 26917981 PMC4762457

[B82] Safiri S. Kolahi A. A. Smith E. Hill C. Bettampadi D. Mansournia M. A. (2020). Global, regional and national burden of osteoarthritis 1990-2017: A systematic analysis of the global burden of disease study 2017. Ann. Rheum. Dis. 79 (6), 819–828. 10.1136/annrheumdis-2019-216515 32398285

[B83] Salminen A. Kaarniranta K. (2012). AMP-activated protein kinase (AMPK) controls the aging process via an integrated signaling network. Ageing Res. Rev. 11 (2), 230–241. 10.1016/j.arr.2011.12.005 22186033

[B84] Schadler P. Lohberger B. Stündl N. Stradner M. H. Glanzer D. Sadoghi P. (2021). The effect of body mass index and metformin on matrix gene expression in arthritic primary human chondrocytes. Cartilage 13 (2), 1004S–1018S. 10.1177/1947603520962558 33025801 PMC8804722

[B85] Snell D. L. Dunn J. A. Jerram K. Hsieh C. J. DeJong G. Hooper G. J. (2021). Associations between comorbidity and quality of life outcomes after total joint replacement. Qual. Life Res. 30 (1), 137–144. 10.1007/s11136-020-02610-6 32816223

[B86] Sokolove J. Lepus C. M. (2013). Role of inflammation in the pathogenesis of osteoarthritis: Latest findings and interpretations. Ther. Adv. Musculoskelet. Dis. 5 (2), 77–94. 10.1177/1759720X12467868 23641259 PMC3638313

[B87] Spicarova D. Nerandzic V. Palecek J. (2014). Update on the role of spinal cord TRPV1 receptors in pain modulation. Physiol. Res. 63 (1), S225–S236. 10.33549/physiolres.932713 24564662

[B88] Taruc-Uy R. L. Lynch S. A. (2013). Diagnosis and treatment of osteoarthritis. Prim. Care 40 (4), 821–836. 10.1016/j.pop.2013.08.003 24209720

[B89] Thomas I. Gregg B. (2017). Metformin; a review of its history and future: From lilac to longevity. Pediatr. Diabetes 18 (1), 10–16. 10.1111/pedi.12473 28052534

[B90] Tian R. Li R. Liu Y. Liu J. Pan T. Zhang R. (2019). Metformin ameliorates endotoxemia-induced endothelial pro-inflammatory responses via AMPK-dependent mediation of HDAC5 and KLF2. Biochim. Biophys. Acta. Mol. Basis Dis. 1865 (6), 1701–1712. 10.1016/j.bbadis.2019.04.009 31002870

[B91] Tsai T. L. Manner P. A. Li W. J. (2013). Regulation of mesenchymal stem cell chondrogenesis by glucose through protein kinase C/transforming growth factor signaling. Osteoarthr. Cartil. 21 (2), 368–376. 10.1016/j.joca.2012.11.001 23151458

[B92] (UKPDS) Group (1998). Intensive blood-glucose control with sulphonylureas or insulin compared with conventional treatment and risk of complications in patients with type 2 diabetes (UKPDS 33). UK Prospective Diabetes Study (UKPDS) Group[J]. Lancet 352 (9131), 837–853. 9742976

[B93] van Lent P. L. Blom A. B. van der Kraan P. Holthuysen A. E. M. VittErs E. vaN RooijeNN. (2004). Crucial role of synovial lining macrophages in the promotion of transforming growth factor beta-mediated osteophyte formation. Arthritis Rheum. 50 (1), 103–111. 10.1002/art.11422 14730606

[B94] van Walsem A. Pandhi S. Nixon R. M. Guyot P. Karabis A. Moore R. A. (2015). Relative benefit-risk comparing diclofenac to other traditional non-steroidal anti-inflammatory drugs and cyclooxygenase-2 inhibitors in patients with osteoarthritis or rheumatoid arthritis: A network meta-analysis. Arthritis Res. Ther. 17 (1), 66. 10.1186/s13075-015-0554-0 25879879 PMC4411793

[B95] Vandewauw I. De Clercq K. Mulier M. Held K. Pinto S. Van Ranst N. (2018). A TRP channel trio mediates acute noxious heat sensing. Nature 555 (7698), 662–666. 10.1038/nature26137 29539642

[B96] Vinik A. I. Nevoret M. L. Casellini C. Parson H. (2013). Diabetic neuropathy. Endocrinol. Metab. Clin. North Am. 42 (4), 747–787. 10.1016/j.ecl.2013.06.001 24286949

[B97] Wang B. Nie J. Wu L. Hu Y. Wen Z. Dong L. (2018). AMPKα2 protects against the development of heart failure by enhancing mitophagy via PINK1 phosphorylation. Circ. Res. 122 (5), 712–729. 10.1161/CIRCRESAHA.117.312317 29284690 PMC5834386

[B98] Wang C. Yang Y. Zhang Y. Liu J. Yao Z. Zhang C. (2019). Protective effects of metformin against osteoarthritis through upregulation of SIRT3-mediated PINK1/Parkin-dependent mitophagy in primary chondrocytes. Biosci. Trends 12 (6), 605–612. 10.5582/bst.2018.01263 30584213

[B99] Wang C. Yao Z. Zhang Y. Yang Y. Liu J. Shi Y. (2020). Metformin mitigates cartilage degradation by activating AMPK/SIRT1-Mediated autophagy in a mouse osteoarthritis model. Front. Pharmacol. 11, 1114. 10.3389/fphar.2020.01114 32792951 PMC7393141

[B100] Wang S. Kobayashi K. Kogure Y. Yamanaka H. Yamamoto S. Yagi H. (2018). Negative regulation of TRPA1 by AMPK in primary sensory neurons as a potential mechanism of painful diabetic neuropathy. Diabetes 67 (1), 98–109. 10.2337/db17-0503 29025860

[B101] Wang Y. Hussain S. M. Wluka A. E. Lim Y. Z. Abram F. Pelletier J. P. (2019). Association between metformin use and disease progression in obese people with knee osteoarthritis: Data from the osteoarthritis initiative-a prospective cohort study. Arthritis Res. Ther. 21 (1), 127. 10.1186/s13075-019-1915-x 31126352 PMC6534888

[B102] Wei Y. Wang Y. Wang Y. Bai L. (2017). Transient receptor potential vanilloid 5 mediates Ca2+ influx and inhibits chondrocyte autophagy in a rat osteoarthritis model. Cell. Physiol. biochem. 42 (1), 319–332. 10.1159/000477387 28535500

[B103] Wojdasiewicz P. Poniatowski Ł. A. Szukiewicz D. (2014). The role of inflammatory and anti-inflammatory cytokines in the pathogenesis of osteoarthritis. Mediat. Inflamm. 2014, 561459. 10.1155/2014/561459 PMC4021678 24876674

[B104] Yan J. Ding D. Feng G. Yang Y. Zhou Y. Ma L. (2022). Metformin reduces chondrocyte pyroptosis in an osteoarthritis mouse model by inhibiting NLRP3 inflammasome activation. Exp. Ther. Med. 23 (3), 222. 10.3892/etm.2022.11146 35222699 PMC8812147

[B105] Zhang M. Liu Y. Huan Z. Wang Y. Xu J. (2020). Metformin protects chondrocytes against IL-1β induced injury by regulation of the AMPK/NF-κ B signaling pathway. Pharmazie 75 (12), 632–636. 10.1691/ph.2020.0762 33303055

[B106] Zhang Y. Vasheghani F. Li Y. H. Blati M. Simeone K. Fahmi H. (2015). Cartilage-specific deletion of mTOR upregulates autophagy and protects mice from osteoarthritis. Ann. Rheum. Dis. 74 (7), 1432–1440. 10.1136/annrheumdis-2013-204599 24651621

[B107] Zhao X. Petursson F. Viollet B. Lotz M. Terkeltaub R. Liu-Bryan R. (2014). Peroxisome proliferator-activated receptor γ coactivator 1α and FoxO3A mediate chondroprotection by AMP-activated protein kinase. Arthritis Rheumatol. 66 (11), 3073–3082. 10.1002/art.38791 25047750 PMC4343036

[B108] Zhou S. Lu W. Chen L. Ge Q. Chen D. Xu Z. (2017). AMPK deficiency in chondrocytes accelerated the progression of instability-induced and ageing-associated osteoarthritis in adult mice. Sci. Rep. 7, 43245. 10.1038/srep43245 28225087 PMC5320548

[B109] Zhuang C. Wang Y. Zhang Y. Xu N. (2018). Oxidative stress in osteoarthritis and antioxidant effect of polysaccharide from angelica sinensis. Int. J. Biol. Macromol. 115, 281–286. 10.1016/j.ijbiomac.2018.04.083 29673954

